# Potential of *Lactobacillus plantarum* IBB3036 and *Lactobacillus salivarius* IBB3154 to persistence in chicken after *in ovo* delivery

**DOI:** 10.1002/mbo3.620

**Published:** 2018-03-25

**Authors:** Tamara Aleksandrzak‐Piekarczyk, Weronika Puzia, Joanna Żylińska, Jarosław Cieśla, Krzysztof A. Gulewicz, Jacek K. Bardowski, Roman K. Górecki

**Affiliations:** ^1^ Institute of Biochemistry and Biophysics Polish Academy of Sciences Warsaw Poland; ^2^ Institute of Bioorganic Chemistry Polish Academy of Sciences Poznań Poland

**Keywords:** *Lactobacillus*, persistence, poultry, prebiotic

## Abstract

The aim of this study was to characterize and compare selected *Lactobacillus* strains originating from different environments (cow milk and hen feces) with respect to their applicative potential to colonize gastrointestinal track of chickens before hatching from an egg. *In vitro* phenotypic characterization of lactobacilli strains included the investigation of the important prerequisites for persistence in gastrointestinal tract, such as a capability to survive in the presence of bile salts and at low pH, enzymatic and sugar metabolic profiles, adhesion abilities, and resistance to osmolytes, temperature, and antibiotics. Regarding the resistance of lactobacilli to most of the various stress factors tested, the milk isolate *Lactobacillus plantarum* IBB3036 showed better abilities than the chicken feces isolate *Lactobacillus salivarius* IBB3154. However, regarding the acidification tolerance and adherence ability, *L. salivarius* IBB3154 revealed better characteristics. Use of these two selected lactobacilli isolates together with proper prebiotics resulted in the preparation of two S1 and S2 bioformulations, which were injected *in ovo* into hen Cobb500 FF fertilized eggs. Furthermore, *in vivo* tests assessing the persistence of *L. plantarum* IBB3036 and *L. salivarius* IBB3154 in the chicken gastrointestinal tract was monitored by PCR‐based classical and quantitative techniques and revealed the presence of both strains in fecal samples collected 3 days after hatching. Subsequently, the number of *L. salivarius* IBB3154 increased significantly in the chicken intestine, whereas the presence of *L. plantarum* IBB3036 was gradually decreased.

## 1 INTRODUCTION

Lactic acid bacteria (LAB) are important autochthonous residents in the chicken gastrointestinal tract (GIT) (Musikasang, Tani, H‐kittikun, & Maneerat, 2009). GITs of birds are colonized by beneficial bacteria in nests when progeny (offspring) are exposed to hen and nest materials (de Oliveira, van der Hoeven‐Hangoor, van de Linde, Montijn, & van der Vossen, 2014). Inasmuch as modern poultry farms prevent chicken contact with hens, the alimentary canals of newly hatched birds could be colonized by bacteria including pathogens present in a hatchery (de Oliveira et al., 2014). Mead (Mead, 1997) showed that lactobacilli inhabit the small intestine and caeca of chickens 1 week after hatching. It has been noted that the colonization of GIT with nonpathogenic microorganisms creates a protective surface against pathogens (Mahroop Ra, Raja, & Mohamed Im, 2009). *In ovo* administration of probiotic bacteria may lead to GIT colonization before the first contact with environmental microorganisms (Villaluenga, Wardeńska, Pilarski, Bednarczyk, & Gulewicz, 2004). The initial microbe inoculation may protect the gut of the chick from pathogenic bacteria by competitive exclusion mechanisms, which are based on bacterial interactions mediated by competition for mucosal adhesion sites and nutrients (Majidzadeh Heravi et al., 2011). Moreover, the initial delivery of desirable bacteria is crucial for processes referring to the development and maturation of the immune system (Di Bartolomeo, Startek, & Van den Ende, 2012). Lactobacilli are natural inhabitants of GIT and are able to ferment various sugars (hexoses, pentoses, and disaccharides) to lactic acid. The lactate‐dependent strong acidification, in addition to bacteriocin production, can influence anti‐pathogen properties of the protective surface to be more efficient and ultimately may prevent the proliferation of undesirable bacteria and fungi. Thus, these phenomena are involved in maintaining the microbiological balance in the intestines (Mahroop Ra et al., 2009). The impact of lactobacilli on the natural balance of the microflora occurs when strains exhibit competitive exclusion abilities, attachment to epithelial cells of the intestine, fermentation of a broad spectrum of sugars including complex sugars, enhancement of the immune system and resistance to inner digestive track conditions such as a low pH and the presence of bile salts (Ding & Shah, 2007). Among the evaluation of probiotic safety, it should be ascertained that any given probiotic strain is not at significant risk with regard to transferable antibiotic resistance genes (Ramirez‐Chavarin, Wacher, Eslava‐Campos, & Perez‐Chabela, 2013). Nurami and Rantal (Nurmi & Rantala, 1973) reported a phenomenon of prevention against Salmonellae infections by oral administration of gut microorganisms in chickens. Moreover, the results of some other studies have evidenced that probiotics may represent an alternative for antibiotics (Majidzadeh Heravi et al., 2011). Additionally, desirable bacteria improve digestive processes and, in parallel, might have an impact on the nutrient intake and growth of farm chickens (Taheri, Moravej, Tabandeh, Zaghari, & Shivazad, 2009). As a result, desirable bacteria can have a positive impact on the feed conversion ratio (FCR), resulting in better meat (weight) gain.

To increase the chance of survival and the persistence of bacteria in GIT, some complex sugars are used as prebiotics (Bednarczyk et al., 2016). Prebiotics are defined as non/low‐digestible food ingredients, which pass undigested into the lower gut where they become available for some colonic bacteria but are not utilized by the majority of the bacteria present in the colon. Lactulose, galactooligosaccharides, fructooligosaccharides, inulin and its hydrolysates, maltooligosaccharides, and starch are the most‐known prebiotics. The ability to ferment prebiotics is mainly associated with the bifidobacteria but also some lactobacilli (Grajek, Olejnik, & Sip, 2005). Since the role of LAB in the feed conversion efficacy and health of the birds was recognized, *Lactobacillus* strains have been extensively studied and used as synbiotics, which refer to a mixture of probiotic and prebiotic compounds (Ehrmann, Kurzak, Bauer, & Vogel, 2002; Patterson & Burkholder, 2003). Their synergistic effects can potentiate the implantation and persistence of desirable bacteria in animal GIT.

Many bioprotective formulations are based on probiotic strains isolated from the natural intestinal microflora of animals. The aim of this study was to characterize selected *Lactobacillus* strains originating from different environments (milk and hen feces) regarding their applicative potential as a beneficial bacteria administered *in ovo*. Various criteria for the selection of a beneficial candidate as well as the assessment of bacterial growth in response to the presence of prebiotic compounds were investigated, resulting in the preparation of two bioactive formulations, which were injected *in ovo* into Cobb500 FF fertilized eggs. The persistence of the two selected *Lactobacillus* strains in the gut was monitored, using a PCR‐based quantitative method showing the presence of both strains in 3‐day fecal samples and the considerable species‐dependent variability over that time.

## 2 MATERIALS AND METHODS

### 2.1 Growth conditions, isolation, and identification of *Lactobacillus* strains

The bacterial strains used in this study are listed in Table 1. *Lactobacillus salivarius* IBB3154 was isolated from chicken stool and identified as described previously (Kobierecka et al., 2015). *Lactobacillus plantarum* strains (IBB3036, IBB3039, IBB3041 and IBB3075) were isolated from raw cow milk samples collected from small, family‐owned farms in the north‐eastern part of Poland. *Lactobacillus* strains were isolated anaerobically at 37°C on MRS‐agar (Merck, Germany). Microscopic observations were performed, using a Biolar C microscope (PZO, Poland).

**Table 1:** Bacterial strains and oligonucleotides used in this study

Species	Strain	Origin	Source or reference
*L. salivarius* a	IBB3154	Chicken feces	(Kobierecka et al., 2015)
*L. plantarum* a	IBB3036 IBB3039 IBB3041 IBB3075	Row cow milk	This study
*L. rhamnosus*	GG	Highly adhesive human intestine isolate	(Segers & Lebeer, 2014)
Primer	Sequence	Product size (bp)	Source or reference
1406R	ACGGGCGGTGTGTRC	1063	(Salama, Sandine, & Giovannoni, 1991)
343F	TACGGGAGGCAGCAG		
16‐1A	GAATCGCTAGTAATCG	500 and 700	(Tannock et al., 1999)
23‐1B	GGGTTCCCCCATTCGGA		
Ls12qF	CTTCGTCCAGCCAAGATAG	207	This study
Ls12qR	GGGATTTGGAGCTGGATATG		
Lp18qF	TCGTACTAACGTCACCATTG	193	This study
Lp18qR	CTAAGGGATGAGGTGATCTTG		
EfGDHqF	CCTGGAGCGATTAACACAC	189	This study
EfGDHqR	CATCCCGCCATCTACAAAG		
EfPTSMqF	GCCATTGCATCGTTTGAC	188	This study
EfPTSMqR	TCTTGTGCTGATTCCATAGAG		
Ls11F	TACAGGTGCTGGAAACGATG	786	This study
Ls11r	TCGGGCATTGTCATCGTTAC		
Ls12F	ATCTGGGCCTTCGAATGTAG	1020	This study
Ls12R	CCTGCTGGTAAAGCAATGTC		
Lp13F	CCCGATGTTTGCAGTACTTG	396	This study
Lp13R	TTATGTACAGCCGGGATTGC		
Lp18F	CAGCTTATGCCGATTCTTGC	600	This study
Lp18R	GAGCTTACTCGAGGAAGGTTTG		

a The IBB strains were banked in a publicly accessible culture collection of PCM (WDCM 106) under numbers PCM 2859, PCM 2860, PCM 2861, PCM 2862, PCM 2863, respectively.

To identify bacterial strains, total DNA was isolated using a Classic Minicolumn kit (A&A Biotechnology, Poland). First, genus‐specific primers (16‐1A and 23‐1B; Table 1) amplifying the intergenic space region between 16S and 23S rDNA were used. For 16S rDNA sequencing, the 1063‐bp fragment of the 16S rDNA gene was amplified by PCR using primers 343F and 1406R (Table 1). Primer synthesis and sequencing of PCR products were performed using the DNA Sequencing & Oligonucleotide Synthesis Service at IBB PAS (Poland) with the Sequencer ABI377 (Applied Biosystems, USA). To identify the closest homologs, the obtained DNA sequences were aligned using the Basic Local Alignment Search Tool (BLAST) (Altschul, Gish, Miller, Myers, & Lipman, 1990). *Lactobacillus* strains were deposited in the IBB PAS Central Collection of Strains (COLIBB, Poland) and Polish Collection of Microorganisms (PCM, Poland) (Table 1). All solutions that required sterilization were autoclaved in the MICROJET microwave autoclave (ENBIO, Poland).

### 2.2 Enzymatic activity and sugar fermentation profiles

The enzymatic activity and sugar fermentation patterns were assessed at least in duplicate using the API^®^ 50 CHL and API^®^ ZYM kits, respectively, following the manufacturer's (BioMerieux, France) protocol. API^®^ ZYM tests 19 different enzymes, whereas API^®^ 50 CH detects the fermentation of 49 different carbon sources. The resulting sugar fermentation pattern was inspected following anaerobic incubation at 37°C after 48 hr.

### 2.3 Growth and spot assays under acid, bile salts, sodium chloride, and temperature stresses

The acid tolerance of *L. plantarum* IBB3036 and *L. salivarius* IBB3154 was investigated in NaCl 0.9% adjusted with 5 M HCl to pH 2.5, 3.5 and 7 (used as a positive control). Briefly, overnight (o/n) bacterial cultures grown in MRS were centrifuged (6000 *g*, 7 min), and the cell pellet was resuspended in saline to avoid the introduction of residual sugars into the test solution. Subsequently, each strain was diluted 100‐fold in test tubes containing saline solution adjusted to different pH values and incubated (37°C; 1.5–3 hr).

To evaluate bile salt tolerance, *L. plantarum* IBB3036 and *L. salivarius* IBB3154 were grown overnight in MRS broth. Subsequently, each strain was diluted 100‐fold in test tubes containing MRS supplemented with different concentrations (0, 0.3, 1%) of ox gall (Sigma‐Aldrich, USA) and incubated (37°C; 1.5–3 hr). In both tests, samples obtained at two time points (1.5 and 3 hr) were collected for enumeration. Viable cell counts were determined by plating on solid MRS and calculated by comparing the common logarithm of the final count after the time points, with different medium supplementation (0%, 0.3%, 1% of ox gall or pH 2.5, 3.5 and 7) and with the common logarithm of the final plate count in pure MRS broth. *Lactobacillus* survival was expressed as “%” and calculated as follows: Na/Nb × 100, where Na = log colony‐forming units (CFU)/ml after incubation and Nb = log CFU/ml before incubation. Each experiment was carried out in three independent repetitions.

To assess the effects of osmolarity, 3 μl of an o/n culture of each *Lactobacillus* strain was spotted on plates with MRS‐agar broth (positive control) and MRS‐agar containing 2, 4, 6 or 8% (w/v) NaCl (Sigma‐Aldrich, USA) and incubated at 37°C for 48 hr. The survival of the bacteria was examined visually by comparison of their growth efficiency on MRS‐agar with their growth on MRS‐agar with different concentrations of NaCl.

The growth of the *Lactobacillus* strains at different temperatures (16, 37 or 45°C) was tested as a spot assay on MRS‐agar plates. Using this approach, 5 μl of o/n culture or its dilutions of each *Lactobacillus* strain was spotted on plates with MRS‐agar and subsequently incubated for 48 hr at 16, 37 or 45°C. The growth of the bacteria was examined visually.

### 2.4 Adhesion assay to bare polystyrene (PS) under static conditions

Bacteria were evaluated for their adhesion ability to bare polystyrene in 96‐well microtiter plates (Thermo Fischer Scientific Nunc A/S, Denmark) using the technique described by (Christensen, Baldassarri, & Simpson, 1995) and modified by Radziwill‐Bienkowska (Radziwill‐Bienkowska et al. 2014). Each strain was tested in three independent experiments, and the results are presented as the means ± standard deviations. Each microtiter plate included the control high‐adhesive *L. rhamnosus* GG (Segers & Lebeer, 2014) and blank wells with PBS (phosphate‐buffered saline). To calculate the adherence ratio of bacteria to PS, the value of absorbance of bacteria was divided by the absorbance of the control sample (PBS only). The bacteria were characterized as strongly adherent (A ≥ 3), moderately adherent (3 > A ≥ 2), weakly adherent (2 > A > 1.5), and nonadherent (A ≤ 1.5).

### 2.5 Antibiotic minimum inhibitory concentration (MIC) determination

E‐test gradient strips of gentamicin, kanamycin, streptomycin, tetracycline, erythromycin, clindamycin, chloramphenicol, ampicillin or vancomycin were used (BioMerieux, France). The choice of nine antibiotics was performed according to the EFSA guidelines on Additives and Products or Substances used in Animal Feed (EFSA Panel on Additives and Products or Substances used in Animal Feed (FEEDAP) 2012). The E‐test was performed following the manufacturer's instructions using LMS‐agar medium composed of MRS and Iso‐Sensitest medium (Oxoid, UK) with weight proportions of 10% and 90%, respectively. The inoculum was prepared by selecting three o/n colonies grown on MRS‐agar and suspending them separately in 0.5 ml of sterile 0.9% saline. Drops of the resulting solutions were added to 5 ml of sterile 0.9% saline to adjust the turbidity to 0.5 according to the McFarland standard. Bacteria were spread on LMS‐agar surface with a swab soaked with the final cell suspension. Subsequently, the strips were laid on the LMS‐agar‐bacteria surface and incubated at 37°C for 48 hr. The MIC was determined from the inhibition curves that intersected the scale on a strip.

### 2.6 *In ovo* administration of *L. plantarum* IBB3036 and *L. salivarius* IBB3154 in chickens

Two bioactive formulations, S1 (*L. salivarius* IBB3154 + Bi^2^tos, Clasado Ltd, representing *trans*galactooligosaccharides) and S2 (*L. plantarum* IBB3036 + lupin Raffinose Family Oligosaccharides, RFOs), were selected based on the microbiological experiments already described (Dunislawska et al., 2017). S2 or S1 bioactive formulations were injected separately *in ovo* into the air chamber of Cobb500 FF fertilized eggs (Cobb Vantress, UK) on day 12 of embryonic development. Based on bench studies, following doses of S1 (10^4^ cfu and 2 mg prebiotic per egg) and S2 (10^5^ cfu and 2 mg prebiotic per egg) were selected for further experiment (Dunislawska et al., 2017).

The experiment was conducted in a commercial hatchery (Piast Grupa, Lewkowiec, Poland). At three time points (3 days and 3 and 6 week after hatching), fecal samples were collected and used for DNA isolation and qPCR.

Injection *in ovo*, hatchability and analyses of embryo mortality were conducted at UTP (Dunislawska et al., 2017).

### 2.7 Identification and quantification of bacteria from chicken feces

Total DNA from fecal samples was isolated using a Stool Minicolumn Kit (A&A Biotechnology, Poland). To detect the DNA of *L. plantarum* IBB3036 or *L. salivarius* IBB3154, multiplex PCR assay was employed, using the primers listed in Table 1. For the detection of *L. salivarius* IBB3154 using the classical PCR method, strain‐specific Ls12F, Ls12R, Ls11F, and Ls11R primers were used. To detect *L. plantarum* IBB3036, strain‐specific Lp13F, Lp13R, Lp18F, and Lp18R primers were used. The qPCR assays were conducted using the PikoReal 96 Real‐Time PCR System (Thermo Scientific, USA) or Light Cycler 480 (Roche, Switzerland). Primers were designed using the Primer Quest programme (http://eu.idtdna.com/PrimerQuest/Home/Index). Each reaction was performed in a reaction mixture containing 1× concentrated Real‐Time 2xHS‐PCR Master Mix SYBR A (A&A Biotechnology, Poland), forward and reverse‐specific primers (100 nM each), DNA template (in three amounts: 7.5, 1.5 and 0.3 ng per well, each in duplicate), and water to a final volume 10 μl.

Reactions were performed with an initial denaturation step (3 min; 95°C) followed by 45–50 cycles of denaturation (15 s; 95°C) and primer annealing‐extension (30 s; 60°C). Fluorescence was read during the annealing‐extension step of each cycle. After cycling, melting point temperature analysis was performed in the range of 60–95°C with temperature increments that were dependent on the apparatus used. The quality of the results was evaluated based on expected C_t_ differences among three cDNA amounts as well as product melting curves. Rare outlying results were omitted in the calculations. Three concentrations of DNA permitted the calculation of individual efficiencies for each primer pair and normalization of all results to one, which was common for all genes and DNA concentrations. The amount of each target gene was calculated by the ΔC_t_ method with a geometric mean of two common genes as a reference (Vandesompele et al., 2002). In this approach, two primer pairs (EfGDHqF‐EfGDHqR and EfPTSMqF‐EfPTSMqR) specific for the *E. faecalis* DNA regions were designed based on sequences obtained from the GenBank database. The primer pairs (Lp18qF‐Lpq18R or Ls12qF‐Ls12qR) dedicated to *L. plantarum* IBB3036 or *L. salivarius* IBB3154*,* respectively, were employed in the qPCR assay. The genomes of *L. plantarum* IBB3036 and *L. salivarius* IBB3154 were sequenced using the shotgun approach and the Illumina MiSeq instrument (data not shown). DNA sequencing and primer synthesis was performed at the DNA Sequencing & Oligonucleotide Synthesis Service at IBB PAS in Warsaw.

## 3 RESULTS

### 3.1 Isolation and identification of *Lactobacillus* species

*L. salivarius* IBB3154 was isolated from chicken stool and identified as described previously (Kobierecka et al., 2015). Macroscopic observations showed that the other 4 isolates characterized in this study appeared as round milky‐white and opaque colonies when grown on MRS agar plates. Under a phase contrast microscope, the bacterial cells appeared mainly as single short rods (*L. plantarum* IBB3036, IBB3041) or rods that formed chains with a maximum of 4 cells (*L. plantarum* IBB3039 and IBB3075). The PCR reaction using *Lactobacillus* genus‐specific primers 16‐1A and 23‐1B generated DNA fragments of the expected size for lactobacilli (500 and 700 bp). Subsequently, the 16S rDNA was sequenced and the homology BLAST searches revealed 99% identity to the 16S rDNA sequences in GenBank of *L. plantarum* strains.

### 3.2 Carbohydrate fermentation profile and enzyme activity

**Figure 1:**
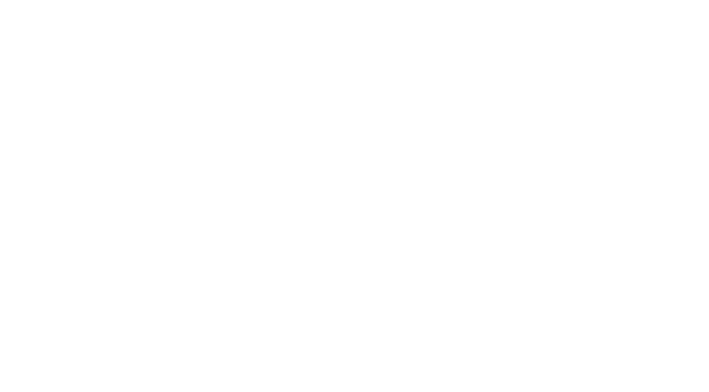
Carbohydrate assimilation capacities and enzyme activities among *Lactobacillus plantarum* and *L. salivarius* strains. Enzyme activity and sugar fermentation ability and efficacy is indicated by different color and size tetragons. The presented carbohydrate assimilation pattern is based only on carbon sources, for which the utilization was variable among species

Among 49 carbohydrates present in the API^®^ 50 CH test, the assimilation of 10 sugars (d‐fructose, d‐galactose, d‐glucose, d‐lactose, d‐maltose, d‐mannitol, d‐mannose, d‐melibiose, d‐sucrose and *N*‐acetylglucosamine) was common for all analyzed *Lactobacillus* spp. (data not shown), whereas the metabolic ability and efficiency of the other 15 carbon sources (amygdaline, arbutin, d‐cellobiose, d‐melezitose, d‐raffinose, d‐ribose, d‐sorbitol, d‐turanose, esculin, gluconic acid, inositol, l‐arabinose, methyl α‐d‐mannopyranoside, salicin and β‐gentiobiose) was species or strain‐dependent (Figure 1). None of the strain utilized 2‐ketogluconate potassium, 5‐ketogluconate potassium, d‐adonitol, d‐arabinose, d‐arabitol, d‐fucose, d‐lyxose, d‐tagatose, d‐trehalose, dulcitol, d‐xylose, glycogen, inulin, l‐arabitol, l‐fucose, l‐rhamnose, l‐sorbose, l‐xylose, methyl‐α‐d‐glucopyranoside, methyl‐β‐d‐xylopyranoside, starch, or xylitol.

A comparison of the two tested species revealed that all *L. plantarum* used a wide range of simple and more complex carbohydrates ranging from 20 (*L. plantarum* IBB3075) up to 24 (*L. plantarum* IBB3036). In contrast, the observed spectrum of sugar metabolism of the *L. salivarius* isolate was much narrower and, among the differentially metabolized sugars, limited solely to d‐raffinose and inositol (Figure 1). In comparison to the *L. plantarum* strains, *L. salivarius* was defective in the metabolism of all β‐glucosides and α‐glucosides, as well as more simple sugars such as d‐ribose, d‐sorbitol, gluconic acid and l‐arabinose.

Regarding oligo and polysaccharide metabolism, all strains of both species were able to metabolize the α‐galactooligosaccharides‐family (αGOS) d‐melibiose [α‐Gal‐(1 → 6)‐Glu] and maltose, the oligosaccharides from the group of α‐glucans, which are the most omnipresent sugars in the intestinal tract of grain‐eating animals (Gänzle & Follador, 2012). However, the metabolism of a trisaccharide, d‐melezitose (α‐Glu‐(1→3)‐β‐Fru‐(2→1)‐α‐Glu), and disaccharide, d‐turanose (α‐Glu‐(1→3)‐α‐Fru), seems to be a common feature among all *L. plantarum* strains tested but is not found in *L. salivarius* IBB3154 (Figure 1). Raffinose family oligosaccharide (RFO) d‐raffinose utilization was limited only to *L. planatrum* IBB3036 and *L. salivarius* IBB3154 (Figure 1), whereas all strains lacked the ability to metabolize d‐trehalose [α‐Glu‐(1 → 1)‐α‐Glu]. In contrast to the ubiquitous oligosaccharide metabolism abilities, the majority of lactobacilli were not amylolytic (Gänzle & Follador, 2012). Indeed, among five strains tested, none showed a capacity for starch metabolism.

Furthermore, the enzymatic profiles of *L. plantarum* IBB3036 and *L. salivarius* IBB3154 were assessed. In both species, negative reactions were observed for more than half of the API^®^ ZYM system enzymes, including alkaline phosphatase, esterase (C4), esterase lipase (C8), lipase (C14), valine arylamidase, cysteine arylamidase, trypsin, α‐chymotrypsin, β‐glucuronidase, α‐mannosidase, and α‐fucosidase. *L. plantarum* IBB3036 and *L. salivarius*. IBB3154 cells showed strong positive acid phosphatase, napthol‐AS‐BI‐phosphohydrolase, α‐galactosidase and β‐galactosidase activities (Figure 1). The activities of four other enzymes (leucine arylamidase, α‐glucosidase, β‐glucosidase, and *N*‐acetyl‐β‐glucosaminidase) were present only in *L. plantarum* IBB3036.

### 3.3 Adhesion properties

**Figure 2:**
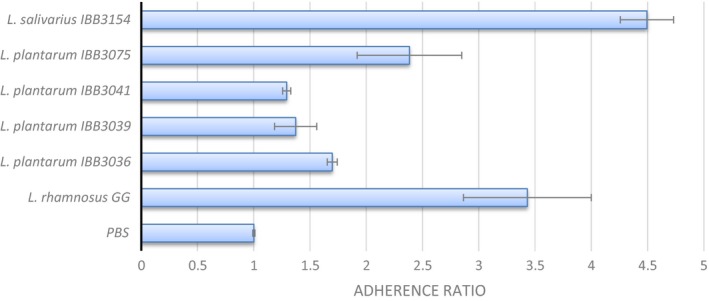
Adhesion of *Lactobacillus* strains to bare PS microtiter plates. Standard deviations (±) from at least three independent experiments are shown as error bars

The adhesion microtiter PS plate assay is a simple method for the preliminary assessment of strain adhesion ability, which usually positively correlates with its adhesiveness to biotic surfaces, as presented, for example, in the study of Aleksandrzak‐Piekarczyk et al. (2016). In this study, we assessed adhesiveness to the PS of 5 *Lactobacillus* strains and compared them with strongly adhesive *L. rhamnosus* GG. Three of four milk‐isolates (*L. plantarum* IBB3036, IBB3039, IBB3041) demonstrated weak or nonadherence, whereas the fourth one (*L. plantarum* 3075) was moderately adherent (Figure 2). Among all species tested, the chicken isolate *L. salivarius* IBB3154 displayed the highest adherence ratio of 4.5 (Figure 2). Remarkably, this strain was even 20% more adherent than control highly adhesive *L. rhamnosus* GG.

### 3.4 Osmotic stress and temperature resistance

**Figure 3:**
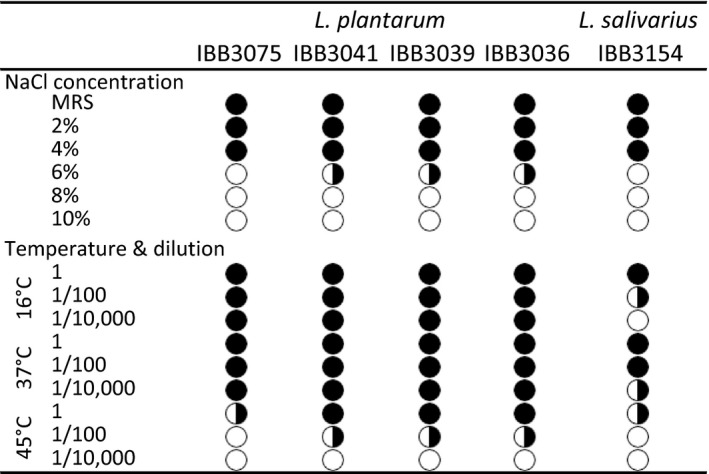
Tolerance of *Lactobacillus* isolates to different concentrations of NaCl and temperatures when assessed by spot assay tests. Black circle—full tolerance, B&W circle—weak tolerance, white circle—lack of tolerance

In this study, the isolated *Lactobacillus* strains showed a similar capacity to survive under an elevated concentration of NaCl, but none of the isolates could grow in 8% NaCl. The strongest resistance (up to 6%) was characteristic of *L. plantarum* IBB3041, IBB3039 and IBB3036, whereas *L. salivarius* IBB3154 and *L. plantarum* IBB3075 revealed a slightly weaker resistance, demonstrating tolerance up to 4% NaCl (Figure 3).

Additionally, the resistance of the five isolated *Lactobacillus* strains to different temperatures was examined. A comparison of the size and density of bacterial spots obtained during strain growth at 37 and 45°C revealed that a temperature of 45°C had a negative effect on the survivability of all *Lactobacillus* strains. The spot‐assay test on an MRS‐agar plate showed that *L. plantarum* IBB3036, IBB3039, and IBB3041 cells were more vital at all temperatures tested than *L. salivarius* IBB3154 cells (Figure 3).

### 3.5 Low pH and bile salt tolerance

**Figure 4:**
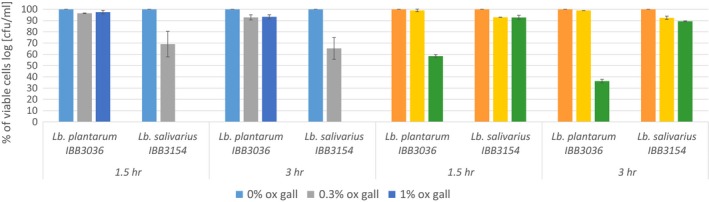
Survival of *Lactobacillus plantarum* IBB3036 and *L. salivarius* IBB3154 after incubation with ox gall or at low pH

The study of acid tolerance showed that cells of both *L. plantarum* IBB3036 and *L. salivarius* IBB3154 strains were vital after an incubation period of 3 hr, at pH 3.5 and pH 2.5. In the case of *L. plantarum* IBB3036, no loss of viability was detected over 3 hr of incubation at pH 3.5 (10^9^ CFU/ml). However, the same strain showed a loss of viability following exposure to pH 2.5 (>10^3^ CFU/ml). The number of *L. salivarius* cells decreased slightly after a 3‐hr incubation at pH 3.5 (from 3 × 10^8^ to 7 × 10^7^ CFU/ml). Nevertheless, *L. salivarius* IBB3154 recorded the strongest acid tolerance because the viability was maintained at a similar level (4 × 10^7^ CFU/ml) even after a 3‐hr exposure to pH 2.5 (Figure 4).

*L. plantarum* IBB3036 and *L. salivarius* IBB3154 strains both survived exposure to 0.3% ox gall. However, under these conditions, the average viability of *L. salivarius* IBB3154 cells was reduced to 65%, whereas the viability of *L. plantarum* IBB3036 declined slightly to 95% compared with control growth in pure MRS broth. The presence of 1% or 2% ox gall had no significant effect on *L. plantarum* IBB3036 growth since the level of viable cells was similar to that observed in 0.3% ox gall. In contrast, the addition of 1% or 2% ox gall completely inhibited the growth of *L. salivarius* IBB3154 (Figure 4).

### 3.6 Antibiotic resistance phenotypes

*L. plantarum* IBB3036 and *L. salivarius* IBB3154 showed a sensitivity to 7 of 9 antibiotics tested in this study (Table 2). Both strains revealed a high level of resistance to vancomycin. Additionally, *L. plantarum* IBB3036 displayed an increased level of resistance to kanamycin in comparison to the cut‐off value (128/64), whereas *L. salivarius* IBB3154 showed increased resistance to streptomycin (96/64). No cross resistance to different aminoglycosides was observed.

**Table 2:** Antibiotic resistance of *Lactobacillus plantarum* IBB3036 and *Lactobacillus salivarius* IBB3154 according to EFSA guidance cut‐off values

Strains	Antibiotics								
	Ampicillin	Vancomycin	Gentamicin	Kanamycin	Streptomycin	Erythromycin	Clindamycin	Tetracycline	Chloramphenicol
	Microbial MIC values (μg/ml)								
*L. plantarum* IBB3036	0.047 (2)^a^	>256 (NR)	1.50 (16)	128 (64)	16 (NR)	0.75 (1)	0.016 (2)	3 (32)	0.75 (8)
*L. salivarius* IBB3154	0.380 (4)^b^	>256 (NR)	0.38 (16)	0.75 (64)	96 (64)	0.75 (1)	0.125 (1)	1 (8)	1.50 (4)

NR, not required due to the intrinsic resistance of bacteria.

a In brackets are listed the cut‐off values for *L. plantarum* published in (EFSA Panel on Additives and Products or Substances used in Animal Feed (FEEDAP) 2012).

b In brackets are listed the cut‐off values for facultative heterofermentative *Lactobacillus* spp. including *L. salivarius* from (EFSA Panel on Additives and Products or Substances used in Animal Feed (FEEDAP) 2012).

### 3.7 Persistence in chicken GIT after *in ovo* administration

All newly designed lactobacilli primer pairs were analyzed, using NCBI/Primer‐BLAST (https://www.ncbi.nlm.nih.gov/tools/primer-blast/). The primer pair specificity was validated using DNA isolated from fecal samples from the chick control group and from chicks from small family‐owned farms. No positive signals were obtained by PCR for either DNA isolated from fecal samples from small family‐owned farms or fecal samples from the control group.

When typing using the classical PCR approach, *L*. *plantarum* IBB3036 was detected in the feces samples of 3‐ and 21‐day chickens with a frequency of 60% and 10%, respectively, and it was not detected in the feces of 42‐day chickens. In contrast, PCR analysis of the microbiome of chickens that had been hatched from eggs injected *in ovo* with S1 revealed *L*. *salivarius* IBB3154 in almost all (95%–100%) samples from all three time points.

**Figure 5:**
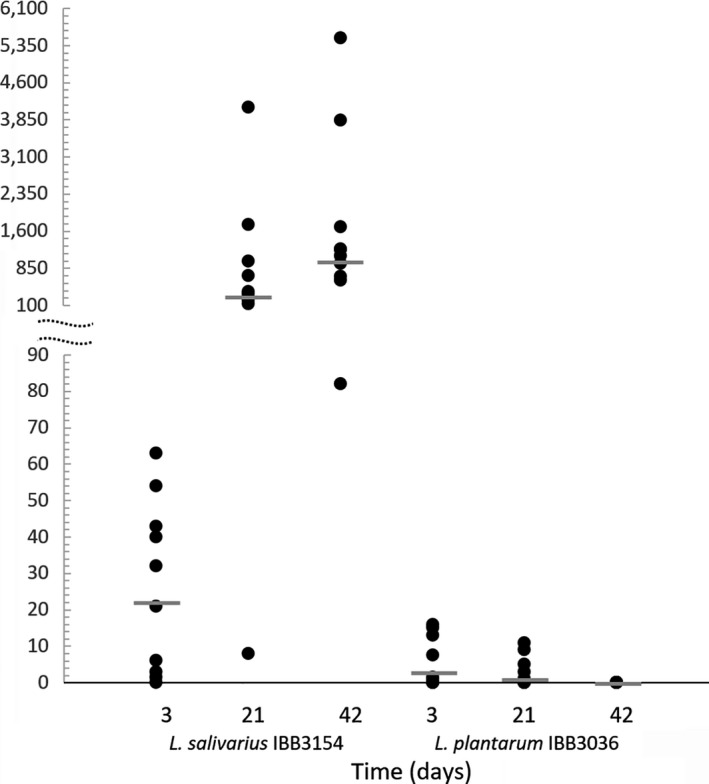
Quantification of *Lactobacillus salivarius* IBB3154 and *L. plantarum* IBB3036 cells present in feces of *in ovo*‐injected chicks. The Y axis values indicate ratio between the number of target lactobacilli and the *E. feacalis* genomes. Dots represent individual chickens, and dashes show median values

The occurrence of strains in chick feces, analyzed by quantitative PCR, is presented as the ratio between numbers of *Lactobacillus* and *E. faecalis* genomes. In microbiome analyses of chickens that have been hatched from eggs injected *in ovo* with S2, the *L*. *plantarum* IBB3036 strain was present in 3‐day chickens at levels 4 times higher than in *E. faecalis*. In the 3‐week chicken group, the median value of the ratio between the number of *L*. *plantarum* IBB3036 and *E. faecalis* genomes declined to 2 and reached zero at the last time point (Figure 5). The results of *L. salivarius* qPCR revealed that feces of 3‐day chickens contained a 26‐fold greater number of *L*. *salivarius* IBB3154 cells than *E. faecalis* cells. At the next two time points, the *L*.* salivarius* IBB3154 median values increased to 350 and 1175, indicating that the number of IBB3154 cells was 10^3^‐fold higher than the number of *E. faecalis* on day 42 of chicken life (Figure 5). Thus, the qPCR results corresponded to the results of the PCR approach used for *Lactobacillus* detection.

## 4 DISCUSSION

Although a reasonable number of well‐characterized probiotic strains are commercially available worldwide, screening for novel strains is still of great interest in general and also from a poultry production perspective. In this process, lactobacilli may be potentially applied as an alternative to antibiotic growth promoters (already banned in the EU) to improve animal health, growth performance and food safety. Strains possessing unique and particular features that may enable health benefits could be derived from intestine as well as the dairy environment. Some authors have promoted the importance of an intestinal origin as a selective criterion for the search for desirable strains, but the expert FAO‐WHO (2006) panel suggested that probiotic activity is more relevant than the source of the bacteria. In fact, the evaluation and selection of dairy LAB isolates for potential use as probiotics has been previously described (Leite et al., 2015; Zheng et al., 2013). Thus, in addition to LAB strains of intestinal origin, those derived from milk could also be used as a source for obtaining novel probiotic strains. Considering the abovementioned information, the aim of this study was to evaluate the beneficial properties of a few *L. planatrum* milk isolates and subsequently test the selected strain persistence in the chick digestive tract after *in ovo* injection in synbiotic form. As a reference for the chick intestine, a *Lactobacillus* isolate was used.

First, we compared the sugar metabolism profile of LAB isolates with a special focus on sugars, which are omnipresent in chicken GIT, and two selected prebiotics (RFOs and Bi^2^tos). Oligosaccharides and higher oligosaccharides are the major carbohydrates in food‐related and intestinal habitats (El‐Fallal, Abou, El‐Sayed, & Omar, 2012; Roberfroid & Slavin, 2000) and their utilization appears to be an ubiquitous feature of lactobacilli (Gänzle & Follador, 2012); however, in this study, it was only characteristic of *L. plantarum* isolates. The observed wide spectrum of sugars utilized by *L. plantarum* strains may be associated with a high versatility of this species as it is commonly found in many different ecological niches (Siezen & van Hylckama Vlieg, 2011) and is reflected in the more than 3‐Mb *L. plantarum* genome, which is among the largest ones of *Lactobacillus* species (http://www.ncbi.nlm.nih.gov/genome/). This significant reduction in the range of sugars metabolized by *L. salivarius* compared to *L. plantarum* may result from the considerably smaller genome size and the ongoing process of its structural minimization of *L. salivarius* species (Casas & Dobrogosz, 2000; Raftis, Salvetti, Torriani, Felis, & O'Toole, 2011). *L. salivarius* IBB3154 inability to metabolize α‐ (d‐melezitose and d‐turanose) and β‐glucosides (amygdaline, arbutin, d‐cellobiose, esculin, salicin and β‐gentiobiose) correlated with its deficiency of α‐ and β‐glucosidase activity. These two glycosyl hydrolases are indispensable for the hydrolysis of α‐ or β‐glycosidic bonds, and the lack of their activity may provide an explanation for the metabolic deficiency of *L. salivarius* IBB3154 α‐ and β‐glucosides. However, other reasons (such as, for example, the lack of proper sugar‐specific transport systems) cannot be excluded. The high β‐galactosidase activity of *L. plantarum* and *L. salivarius* cells correlated with lactose and GOS hydrolysis.

The high level of leucine arylamidase was only present in *L. plantarum* IBB3036. Lactobacilli, as heterotrophic bacteria, can degrade and modify a large variety of plant polymers or metabolites containing amino acids. The activity of leucine arylamidase is a good marker of the proteolytic activity of bacteria as this enzyme is a member of the metallopeptidase group that removes N‐terminal l‐leucine from peptide substrates (Mudryk & Podgorska, 2006).

NaCl is an inhibitory substance, which by acting on the loss of cells membrane and tension turgor pressure, may negatively influence the growth of some bacteria (Liu, Asmundson, Gopal, Holland, & Crow, 1998). The ability to resist its presence is one of the essential natural attributes of desirable strains because to survive and grow in the gastrointestinal tract, bacteria must adapt to an environment with an osmolarity equivalent to 0.3 M NaCl (Chowdhury, Sahu, & Das, 1996). These study results suggest that all analyzed strains could withstand the NaCl osmolarity in the animal GIT and could resist technological processing because they were all tolerant to high, up to 4%–6%, NaCl concentrations, which can be present in pelleted or dried animal food.

Adhesion to mucosa is essential for bacterial persistence in the host. The ability to fix itself to the mucosal layer allows bacteria to avoid being swept away by peristaltic movement (Fernandez, Boris, & Barbes, 2003). It is also postulated that highly adhesive probiotic bacteria have the greatest beneficial effects on host health and should, at least transiently, colonize the host gut (FAO‐WHO 2006). Among the lactobacilli tested during this study, the greatest adherence was noted in strains of intestinal origin, whereas this ability was considerably reduced in milk isolates. According to the data available in the literature, efficient adhesiveness of intestinal isolates does not seem to be the rule because many strains of this origin do not display any ability to adhere (Kobierecka et al., 2017), however, many strains isolated from environments other than the intestine can be effective binders (Douillard et al., 2013).

Tolerance to bile salts and a low pH is essential for bacteria to survive in GIT because they are most commonly orally delivered in a feed system. In comparison to mammals, the alimentary canal of chickens is shorter. The time required for feed to pass through the entire gastrointestinal tract can be as short as 2.5 hr (Duke, 1977; Jin, Ho, Abdullah, & Jalaludin, 1998). Most bacteria do not survive at low pH values, and thus the acidic conditions of the chick crop (pH 4.5), proventriculus (pH 4.4) and gizzard (pH 2.6) could have adverse effects on their well‐being (Pond, 2005). Among bacteria, the lactobacilli are an unique case, with moderate or good resistance up to approximately pH 3, and a marginal decrease in survival under severe acidic conditions (Ding & Shah, 2007; Jin et al., 1998; Taheri et al., 2009). Our results confirm this trend because the exposure of lactobacilli to pH 2.5 showed a slight loss in viability of *L. salivarius* IBB3154 (to 90% of log CFU/ml), whereas the viability of *L. plantarum* IBB3036 was reduced to approximately 33% of the log CFU/ml after 3 hr of incubation, indicating that these bacteria were fully adapted to survive in the chicken alimentary canal.

Bacteria are generally sensitive to bile salts, but some lactobacilli are able to cope with these adverse conditions. In this study, the amount of viable *L. plantarum* IBB3036 strain cells remained essentially steady after 3 hr of incubation with 1% ox gall, and their vitality differed less than 10% in comparison to *L. plantarum* IBB3036 strain cells incubated without ox gall. *L. salivarius* IBB3154 was completely sensitive to this concentration of ox gall. Interestingly, the high tolerance to ox gall of the *L. plantarum* IBB3036 strain, which was originally isolated from milk, was not expected.

Prebiotics are defined as nondigestible food and feed supplements that have beneficial effects on the host by stimulating the growth of desirable bacteria in GIT. Monogastric animals including poultry are not able to break down RFOs and GOS. This study showed that *L. plantarum* IBB3036 and *L. salivarius* IBB3154 were able to hydrolyze both RFOs and GOS, indicating that both prebiotics may enhance bacterial survival in the chicken embryo.

The common usage of antibiotics in the poultry industry was banned (Mahroop Ra et al., 2009); however, knowledge concerning antimicrobial susceptibility is crucial to avoid the introduction of exogenous sources of resistance determinants. According to our results, *L. plantarum* IBB3036 and *L. salivarius* IBB3154 are not considered to harbor antibiotic resistance determinants, although they have higher levels of resistance to vancomycin and streptomycin. It is well known that *Lactobacillus* strains have an intrinsic resistance to vancomycin due to the absence of the appropriate cell wall precursor target. Remarkably, a higher level of resistance to aminoglycoside antibiotics is also considered to represent an intrinsic resistance in lactobacilli and is attributed to the lack of cytochrome‐mediated electron transport (Hummel, Hertel, Holzapfel, & Franz, 2007).

Although most *Lactobacillus* strains only transiently colonize chicken gastrointestinal chicken tracts (Spivey, Dunn‐Horrocks, & Duong, 2014; Stephenson, Moore, & Allison, 2010), the results of this study indicate that *L. salivarius* IBB3154 was able to persist and even to significantly increase in number in the chicken intestine after a single *in ovo* inoculation and within the entire production period. In contrast, *L. plantarum* IBB3036 was detectable at low levels on the third day of the chicks’ life and was virtually undetectable during the last stage of the experiment (day 42). Such a severe discrepancy between both strains was unexpected because they were characterized by similar probiotic features. Moreover, in some aspects, such as the range of sugar metabolism and tolerance to bile salts, NaCl and elevated temperatures, *L. plantarum* IBB3036 showed better capabilities than *L. salivarius* IBB3154 to withstand the harsh environmental conditions of the chicken GIT. Regarding two other probiotic features, tolerance to acidification and adherence ability, *L. salivarius* IBB3154 showed better performance. However, the higher acid resistance of *L. salivarius* IBB3154 cannot be a reason for the pronounced persistence of this strain because the bacteria were introduced by *in ovo* injection and thus were not exposed to the acidic conditions of the chick GIT. Thus, according to our findings, the main reason for the lack of *L. plantarum* IBB3036 persistence may be its poor adherence ability, which led to progressive strain removal by peristalsis and/or its exclusion by other resident gut microorganisms (such as, presumably, *L. salivarius* IBB3154). In contrast, the strong adherence abilities of *L. salivarius* IBB3154 allowed its survival and extensive proliferation in the chick GIT. Confirmation of whether this phenomenon is indeed important for *L. salivarius* IBB3154 adherence abilities and/or other mechanisms requires further studies. Nevertheless, *L. salivarius* IBB3154 seems to have potential for modulation of the GIT microbiota due to the adherence ability and *in ovo* delivery into the chicken embryo, which extends the effective time of action to the prehatching period.

## CONFLICT OF INTEREST

The authors declare that they have no conflict of interest.

## ETHICAL STATEMENT

Animal use was approved by the Local Ethical Committee for Animal Experimentation, University of Sciences and Technology, Bydgoszcz, Poland, on July 13, 2012, no 36/2012.

## References

[mbo3620-bib-0001] Aleksandrzak‐Piekarczyk, T. , Koryszewska‐Bagińska, A. , Grynberg, M. , Nowak, A. , Cukrowska, B. , Kozakova, H. , & Bardowski, J. (2016). Genomic and functional characterization of the unusual pLOCK 0919 plasmid harboring the *spaCBA* pili cluster in *Lactobacillus casei* LOCK 0919. Genome Biology and Evolution, 8, 202–217. 10.1093/gbe/evv247 PMC4758243 26637469

[mbo3620-bib-0002] Altschul, S. F. , Gish, W. , Miller, W. , Myers, E. W. , & Lipman, D. J. (1990). Basic local alignment search tool. Journal of Molecular Biology, 215, 403–410. 10.1016/S0022-2836(05)80360-2 2231712

[mbo3620-bib-0003] Bednarczyk, M. , Stadnicka, K. , Kozłowska, I. , Abiuso, C. , Tavaniello, S. , Dankowiakowska, A. , … Maiorano, G. (2016). Influence of different prebiotics and mode of their administration on broiler chicken performance. Animal, 10, 1271–1279. 10.1017/S1751731116000173 26936310

[mbo3620-bib-0004] Casas, I. A. , & Dobrogosz, W. J. (2000). Validation of the probiotic concept: *Lactobacillus reuteri* confers broad‐spectrum protection against disease in humans and animals. Microbial Ecology in Health and Disease, 12, 247–285. 10.1080/08910600050216246-1

[mbo3620-bib-0005] Chowdhury, R. , Sahu, G. K. , & Das, J. (1996). Stress response in pathogenic bacteria. Journal of Biosciences, 21, 149–160. 10.1007/BF02703105

[mbo3620-bib-0006] Christensen, G. D. , Baldassarri, L. , & Simpson, W. A. (1995). Methods for studying microbial colonization of plastics. Methods in Enzymology, 253, 477–500. 10.1016/S0076-6879(95)53040-1 7476410

[mbo3620-bib-0007] de Oliveira, J. E. , van der Hoeven‐Hangoor, E. , van de Linde, I. B. , Montijn, R. C. , & van der Vossen, J. M. B. M. (2014). In ovo inoculation of chicken embryos with probiotic bacteria and its effect on posthatch *Salmonella* susceptibility. Poultry Science, 93, 818–829. 10.3382/ps.2013-03409 24706958

[mbo3620-bib-0008] Di Bartolomeo, F. , Startek, J. B. , & Van den Ende, W. (2012). Prebiotics to fight diseases: Reality or fiction? Phytotherapy Research, 27, 1457–1473. 10.1002/ptr.4901 23280537

[mbo3620-bib-0009] Ding, W. K. , & Shah, N. P. (2007). Acid, bile, and heat tolerance of free and microencapsulated probiotic bacteria. Journal of Food Science, 72, M446–M450. 10.1111/j.1750-3841.2007.00565.x 18034741

[mbo3620-bib-0010] Douillard, F. P. , Ribbera, A. , Kant, R. , Pietila, T. E. , Jarvinen, H. M. , Messing, M. , … de Vos, W. M. (2013). Comparative genomic and functional analysis of 100 *Lactobacillus rhamnosus* strains and their comparison with strain GG. PLoS Genetics, 9, e1003683 10.1371/journal.pgen.1003683 23966868 PMC3744422

[mbo3620-bib-0011] Duke, G. E. (1977). Avian digestion. In Dukes’ physiology of domestic animals, 19th ed. Ithaca, NY: Cornell University Press.

[mbo3620-bib-0012] Dunislawska, A. , Slawinska, A. , Stadnicka, K. , Bednarczyk, M. , Gulewicz, P. , Jozefiak, D. , & Siwek, M. (2017). Synbiotics for broiler chickens—*in vitro* design and evaluation of the influence on host and selected microbiota populations following *in ovo* delivery. PLoS ONE, 12, e0168587 10.1371/journal.pone.0168587 28045927 PMC5207659

[mbo3620-bib-0013] EFSA Panel on Additives and Products or Substances used in Animal Feed (FEEDAP) (2012). Guidance on the assessment of bacterial susceptibility to antimicrobials of human and veterinary importance: Guidance on the assessment of bacterial antimicrobial susceptibility. EFSA Journal, 10, 2740 10.2903/j.efsa.2012.2740

[mbo3620-bib-0014] Ehrmann, M. A. , Kurzak, P. , Bauer, J. , & Vogel, R. F. (2002). Characterization of lactobacilli towards their use as probiotic adjuncts in poultry. Journal of Applied Microbiology, 92, 966–975. 10.1046/j.1365-2672.2002.01608.x 11972703

[mbo3620-bib-0015] El‐Fallal, A. , Abou, M. , El‐Sayed, A. , & Omar, N. (2012) Starch and microbial α‐amylases: From concepts to biotechnological applications In: C‐F C. (Ed.), Carbohydrates – Comprehensive Studies on Glycobiology and Glycotechnology (pp. 459–488). Rijeka, Croatia: InTech.

[mbo3620-bib-0016] FAO‐WHO (2006) Probiotics in food. Health and nutritional properties and guidelines for evaluation.

[mbo3620-bib-0017] Fernandez, M. F. , Boris, S. , & Barbes, C. (2003). Probiotic properties of human lactobacilli strains to be used in the gastrointestinal tract. Journal of Applied Microbiology, 94, 449–455. 10.1046/j.1365-2672.2003.01850.x 12588553

[mbo3620-bib-0018] Gänzle, M. G. , & Follador, R. (2012). Metabolism of oligosaccharides and starch in lactobacilli: A review. Frontiers in Microbiology, 3, 340 10.3389/fmicb.2012.00340 23055996 PMC3458588

[mbo3620-bib-0019] Grajek, W. , Olejnik, A. , & Sip, A. (2005). Probiotics, prebiotics and antioxidants as functional foods. Acta Biochimica Polonica, 52, 665–671. 16086074

[mbo3620-bib-0020] Hummel, A. S. , Hertel, C. , Holzapfel, W. H. , & Franz, C. M. A. P. (2007). Antibiotic resistances of starter and probiotic strains of lactic acid bacteria. Applied and Environment Microbiology, 73, 730–739. 10.1128/AEM.02105-06 PMC1800751 17122388

[mbo3620-bib-0021] Jin, L. Z. , Ho, Y. W. , Abdullah, N. , & Jalaludin, S. (1998). Acid and bile tolerance of *Lactobacillus* isolated from chicken intestine. Letters in Applied Microbiology, 27, 183–185. 10.1046/j.1472-765X.1998.00405.x 9750324

[mbo3620-bib-0022] Kobierecka, P. A. , Wyszyńska, A. K. , Aleksandrzak‐Piekarczyk, T. , Kuczkowski, M. , Tuzimek, A. , Piotrowska, W. , … Jagusztyn‐Krynicka, E. K. (2017). In vitro characteristics of *Lactobacillus* spp. strains isolated from the chicken digestive tract and their role in the inhibition of *Campylobacter* colonization. MicrobiologyOpen, 6, e00512 10.1002/mbo3.512 PMC5635155 28736979

[mbo3620-bib-0023] Kobierecka, P. , Wyszyńska, A. , Maruszewska, M. , Wojtania, A. , Żylińska, J. , Bardowski, J. , & Jagusztyn‐Krynicka, E. K. (2015). Lactic acid bacteria as a surface display platform for *Campylobacter jejuni* antigens. Journal of Molecular Microbiology and Biotechnology, 25, 1–10. 10.1159/000368780 25662187

[mbo3620-bib-0024] Leite, A. M. O. , Miguel, M. A. L. , Peixoto, R. S. , Ruas‐Madiedo, P. , Paschoalin, V. M. F. , Mayo, B. , & Delgado, S. (2015). Probiotic potential of selected lactic acid bacteria strains isolated from Brazilian kefir grains. Journal of Dairy Science, 98, 3622–3632. 10.3168/jds.2014-9265 25841972

[mbo3620-bib-0025] Liu, S.‐Q. , Asmundson, R. V. , Gopal, P. K. , Holland, R. , & Crow, V. L. (1998). Influence of reduced water activity on lactose metabolism by *Lactococcus lactis* subsp. *cremoris* at different pH values. Applied and Environment Microbiology, 64, 2111–2116. 10.1128/aem.64.6.2111-2116.1998 PMC106286 9603822

[mbo3620-bib-0026] Mahroop Ra, M. M. , Raja, A. , & Mohamed Im, M. (2009). *Lactobacillus* as a probiotic feed for chickens. International Journal of Poultry Science, 8, 763–767. 10.3923/ijps.2009.763.767

[mbo3620-bib-0027] Majidzadeh Heravi, R. , Kermanshahi, H. , Sankian, M. , Nassiri, M. , Heravi Moussavi, A. , Roozbeh Nasiraii, L. , & Varasteh, A. (2011). Screening of lactobacilli bacteria isolated from gastrointestinal tract of broiler chickens for their use as probiotic. African Journal of Microbiology Research, 5, 1858–1868.

[mbo3620-bib-0028] Mead, G. (1997). Bacteria in the gastrointestinal tract of birds In Mackie R. I., White B. A., & Isaacson R. E. (Eds.), Gastrointestinal microbiology (pp. 216–240). New York, NY: Chapman and Hall 10.1007/978-1-4757-0322-1

[mbo3620-bib-0029] Mudryk, Z. , & Podgorska, B. (2006). Enzymatic activity of bacterial strains isolated from marine beach sediments. Polish Journal of Environmental Studies, 15, 441–448.

[mbo3620-bib-0030] Musikasang, H. , Tani, A. , H‐kittikun, A. , & Maneerat, S. (2009). Probiotic potential of lactic acid bacteria isolated from chicken gastrointestinal digestive tract. World Journal of Microbiology and Biotechnology, 25, 1337–1345. 10.1007/s11274-009-0020-8

[mbo3620-bib-0031] Nurmi, E. , & Rantala, M. (1973). New aspects of *Salmonella* infection in broiler production. Nature, 241, 210–211. 10.1038/241210a0 4700893

[mbo3620-bib-0032] Patterson, J. , & Burkholder, K. (2003). Application of prebiotics and probiotics in poultry production. Poultry Science, 82, 627–631. 10.1093/ps/82.4.627 12710484

[mbo3620-bib-0033] Pond, W. G. (ed) (2005). Basic animal nutrition and feeding (5th ed.). Hoboken, NJ: Wiley.

[mbo3620-bib-0300] Radziwill‐Bienkowska, J. M. , Zochowska, D. , Bardowski, J. , Mercier‐Bonin, M. , Kowalczyk, M. (2014). *Lactococcus lactis* IBB477 presenting adhesive and muco‐adhesive properties as a candidate carrier strain for oral vaccination against influenza virus. Acta Biochimica Polonica, 61, 603–607. 25210718

[mbo3620-bib-0034] Raftis, E. J. , Salvetti, E. , Torriani, S. , Felis, G. E. , & O'Toole, P. W. (2011). Genomic diversity of *Lactobacillus salivarius* . Applied and Environment Microbiology, 77, 954–965. 10.1128/AEM.01687-10 PMC3028724 21131523

[mbo3620-bib-0035] Ramirez‐Chavarin, M. , Wacher, C. , Eslava‐Campos, C. , & Perez‐Chabela, M. (2013). Probiotic potential of thermotolerant lactic acid bacteria strains isolated from cooked meat products. International Food Research Journal, 2(20), 991–1000.

[mbo3620-bib-0036] Roberfroid, M. , & Slavin, J. (2000). Nondigestible oligosaccharides. Critical Reviews in Food Science and Nutrition, 40, 461–480. 10.1080/10408690091189239 11186236

[mbo3620-bib-0037] Salama, M. , Sandine, W. , & Giovannoni, S. (1991). Development and application of oligonucleotide probes for identification of *Lactococcus lactis* subsp. *cremoris* . Applied and Environment Microbiology, 57, 1313–1318. 10.1128/aem.57.5.1313-1318.1991 PMC182948 1713027

[mbo3620-bib-0038] Segers, M. , & Lebeer, S. (2014). Towards a better understanding of *Lactobacillus rhamnosus* GG ‐ host interactions. Microbial Cell Factories, 13, S7 10.1186/1475-2859-13-S1-S7 25186587 PMC4155824

[mbo3620-bib-0039] Siezen, R. J. , & van Hylckama Vlieg, J. E. (2011). Genomic diversity and versatility of *Lactobacillus plantarum*, a natural metabolic engineer. Microbial Cell Factories, 10, 1–13. 10.1186/1475-2859-10-S1-S3 21995294 PMC3271238

[mbo3620-bib-0040] Spivey, M. A. , Dunn‐Horrocks, S. L. , & Duong, T. (2014). Epithelial cell adhesion and gastrointestinal colonization of *Lactobacillus* in poultry. Poultry Science, 93, 2910–2919. 10.3382/ps.2014-04076 25239531

[mbo3620-bib-0041] Stephenson, D. P. , Moore, R. J. , & Allison, G. E. (2010). *Lactobacillus* strain ecology and persistence within broiler chickens fed different diets: Identification of persistent strains. Applied and Environment Microbiology, 76, 6494–6503. 10.1128/AEM.01137-10 PMC2950442 20693442

[mbo3620-bib-0042] Taheri, H. R. , Moravej, H. , Tabandeh, F. , Zaghari, M. , & Shivazad, M. (2009). Screening of lactic acid bacteria toward their selection as a source of chicken probiotic. Poultry Science, 88, 1586–1593. 10.3382/ps.2009-00041 19590072

[mbo3620-bib-0043] Taheri, H. , Tabandeh, F. , Moravej, H. , Zaghari, M. , Shivazad, M. , & Shariati, P. (2009). Potential probiotic of *Lactobacillus johnsonii* LT171 for chicken nutrition. African Journal of Biotechnology, 8, 5833–5837.

[mbo3620-bib-0044] Tannock, G. W. , Tilsala‐Timisjarvi, A. , Rodtong, S. , Ng, J. , Munro, K. , & Alatossava, T. (1999). Identification of *Lactobacillus* isolates from the gastrointestinal tract, silage, and yoghurt by 16S‐23S rRNA gene intergenic spacer region sequence comparisons. Applied and Environment Microbiology, 65, 4264–4267. 10.1128/aem.65.9.4264-4267.1999 PMC99775 10473450

[mbo3620-bib-0045] Vandesompele, J. , De Preter, K. , Pattyn, F. , Poppe, B. , Van Roy, N. , De Paepe, A. , & Speleman, F. (2002). Accurate normalization of real‐time quantitative RT‐PCR data by geometric averaging of multiple internal control genes. Genome Biology, 3, RESEARCH0034. 12184808 10.1186/gb-2002-3-7-research0034 PMC126239

[mbo3620-bib-0046] Villaluenga, C. M. , Wardeńska, M. , Pilarski, R. , Bednarczyk, M. , & Gulewicz, K. (2004). Utilization of the chicken embryo model for assessment of biological activity of different oligosaccharides. Folia Biologica (Praha), 52, 135–142. 10.3409/1734916044527502 19058551

[mbo3620-bib-0047] Zheng, Y. , Lu, Y. , Wang, J. , Yang, L. , Pan, C. , & Huang, Y. (2013). Probiotic properties of Lactobacillus strains isolated from Tibetan kefir grains. PLoS ONE, 8, e69868 10.1371/journal.pone.0069868 23894554 PMC3718794

